# A comprehensive profiling of T- and B-lymphocyte receptor repertoires from a Chinese-origin rhesus macaque by high-throughput sequencing

**DOI:** 10.1371/journal.pone.0182733

**Published:** 2017-08-16

**Authors:** Longfei Fu, Xinyang Li, Wei Zhang, Changxi Wang, Jinghua Wu, Huanming Yang, Jian Wang, Xiao Liu

**Affiliations:** 1 BGI Education Center, University of Chinese Academy of Sciences, Shenzhen, China; 2 BGI-Shenzhen, Shenzhen, China; 3 China National Genebank, BGI-Shenzhen, Shenzhen, China; 4 James D. Watson Institute of Genome Science, Hangzhou, China; Monash University, Australia, AUSTRALIA

## Abstract

Due to the close genetic background, high similarity of physiology, and susceptibility to infectious and metabolic diseases with humans, rhesus macaques have been widely used as an important animal model in biomedical research, especially in the study of vaccine development and human immune-related diseases. In recent years, high-throughput sequencing based immune repertoire sequencing (IR-SEQ) has become a powerful tool to study the dynamic adaptive immune responses. Several previous studies had analyzed the responses of B cells to HIV-1 trimer vaccine or T cell repertoire of rhesus macaques using this technique, however, there are little studies that had performed a comprehensive analysis of immune repertoire of rhesus macaques, including T and B lymphocytes. Here, we did a comprehensive analysis of the T and B cells receptor repertoires of a Chinese rhesus macaque based on the 5’—RACE and IR-SEQ. The detailed analysis includes the distribution of CDR3 length, the composition of amino acids and nucleotides of CDR3, V, J and V-J combination usage, the insertion and deletion length distribution and somatic hypermutation rates of the framework region 3 (FR3). In addition, we found that several positions of FR3 region have high mutation frequencies, which may indicate the existence of new genes/alleles that have not been discovered and/or collected into IMGT reference database. We believe that a comprehensive profiling of immune repertoire of rhesus macaque will facilitate the human immune-related diseases studies.

## Introduction

T cells and B cells are the two major lymphocytes in vertebrates’ immune system and play important roles in recognizing foreign antigens by their special transmembrane receptor proteins (T cell receptor or TCR for T cell and B cell receptor or BCR for B cell). TCR is heterodimeric molecules and more than 95% of TCR belong to the αβ type that consist of a α chain and a β chain. BCR is tetramer consisting of two identical light chains (IGL or IGK) and two identical heavy chains (IGH) [1]. The beta chain of TCR and the heavy chain of BCR are made up of a Variable (V), a Diversity (D), a Joining (J) and a Constant (C) genes, whereas the alpha chain of TCR and the light chains of BCR do not have the Diversity (D) gene. In human, there are about 52 TRBV, 2 TRBD and 13 TRBJ genes and about 40 IGHV, 23 IGHD and 6 IGHJ genes [2]. According to the theoretical estimate, there are ~10^13^ unique TCR and ~10^17^ unique BCR within one individual’s peripheral blood [3]. This extremely diversified TCR and BCR pool are primarily generated by the somatic rearrangement of the germline V(D)J genes, then random trimming and addition of non-template nucleotides at the V(D)J junctional sites, combinatorial pairing of different chains (for example, alpha and beta chains for TCR) and somatic hypermutation (for B cells only) further increase the diversity of TCR and BCR dramatically [4, 5]. The CDR3 (complementarity-determining region three) is generated by the recombination of V(D)J genes and is the most diverse region that contacts directly with the peptide-MHC complex.

The Indian-origin rhesus macaque (*Macaca mulatta mulatta*) and Chinese-origin rhesus macaque (*Macaca mulatta lasiota*) are two different subspecies of the Macaca genus, and both of these two subspecies are commonly used as an ideal animal model to study the pathogenesis of human diseases in which T and B lymphocytes serve key roles [6–10]. Therefore, profiling this nonhuman primate’s immune repertoire is very useful and important. Previous works has identified many genes and alleles from TCR’s beta chain and BCR’s heavy and light chains in rhesus macaques, which provides a basis for the immune repertoire study of rhesus macaque [11–16]. At present, the IMGT/GENE-DB still does not have the TRAV and TRAJ reference genes of rhesus macaque, fortunately, in 2017, Greene et al extracted 50 TRAV and 60 TRAJ genes from published rhesus macaque (these TRAV and TRAJ genes were termed as “GJ germline gene database” hereafter) [17, 18]. Thus, we have a relative complete germline gene database, including TRA and TRB genes for TCR and IGK/IGL and IGH genes for BCR, for rhesus macaque for the first time, and we can then do a comprehensive immune repertoire analysis based on this complete database.

The target sequence in immune repertoire sequencing is largely focused on the CDR3, especially in the TCR repertoire sequencing for that a T cell clone can be represented approximatively by a distinct CDR3 (nucleotide level). Whereas a relative complete V sequence is benefit for the BCR repertoire analysis duo to the existence of somatic hypermutation (SHM). In humans, the CDR3 length of TRBV mainly ranges from 10 to 14 amino acids [19]. Li Z et al. found that the length of CDR3 of TRBV of rhesus macaques ranges from 10 to 16 amino acids and 84% of the CDR3 ranged from 11 to 13 amino acids. Thus, the distribution of CDR3 length of TRBV is comparable between humans and rhesus macaques [20]. Up to now, there are mainly two experimental techniques for the sequencing library construction. One method is multiplex PCR (MPCR) and another is Rapid Amplification of cDNA ends (RACE). Both of these two methods are widely used in immune repertoire sequencing (IR-SEQ) [21–24].

Although this powerful IR-SEQ technique has been widely used to characterize the features of TCR/BCR repertoires in patients with diseases in which T/B cells play key roles, but the papers of immune repertoire sequencing on rhesus macaque are still limited. At present, the extensive use of this technique on model animals is limited to mice and zebrafish. As an important animal model in biomedical research, a comprehensive immune repertoire of rhesus macaque, including both TCR and BCR, might provide useful information from a new perspective to facilitate the human diseases research. In this study, we used 5’ RACE method combined with high-throughput sequencing to profile a Chinese-origin rhesus macaque’s T and B cells receptor repertoires and found that the usage of V, J and V-J combination are not equal among TRA, TRB, IGH, IGK and IGL repertoires.

## Materials and methods

### Animals and ethics

In this study, a single Chinese-origin rhesus macaque was used and this healthy macaque (female, five-year-old) was fed in Hua Nan Zoo of Guangzhou (Guangzhou city, Guangdong province of China). This macaque was housed in a spacious cottage (with transparent glass, wire-mesh door and climbable branches) allowing social interactions with other macaques under controlled conditions of temperature, humidity and light. The fresh fruit and vegetables and clean water were provided daily by their care stuff. Before collecting blood, we had confirmed that the physical condition of this macaque is well, and without any specific antigen exposure history in the past six months. All blood collection was performed under gentle fixation and all efforts were made to minimize suffering. This study has been reviewed and approved by the Bioethics and Biological Safety Review Committee of BGI-Shenzhen (Permit Number is: BGI-IRB 14052).

### Blood sampling and RNA extraction

All blood collection was performed subcutaneously from the left hind limb under gentle fixation and all efforts were made to minimize suffering. Food rewards was given after fixation and after the procedure. Now this rhesus still lives in the zoo, healthily. Total 5ml peripheral blood was collected from this macaque at one time, and peripheral blood mononuclear cells (PBMCs) were immediately isolated from peripheral blood using Ficoll-Paque (GE Healthcare) gradient centrifugation. The total RNA was extracted according to the manufacturer’s protocol (Invitrogen). The RNA concentration and integrity were determined on an Agilent Bioanalyzer 2100 (Agilent).

### Sequencing library construction and sequencing

The 5’-RACE technologies were adopted to amplify the CDR3. S1 Fig is used to illustrate the whole library construction (Procedure 1–5, refer to the instruction manual of Invitrogen, https://www.thermofisher.com/order/catalog/product/18374058). The total RNA was used for the reverse transcription (RT) with a set of primers specific to the first constant region (CH1, Primers 1–10, **S1 Table**). The number of the primer ID represents the 5’-end annealing positons of CH1. The capital letter ‘B’ represents the biotin labeled primers. In detail, 2ug RNA was used for RT for each repertoire construction. Besides, the IGHM/G/E/A/D multiplex primers were used for the IGH repertoire; the IGKC/IGLC/CRLC multiplex primers were used for the IGK and IGL repertoire; the TRAC and TRBC primers were separately used for the TCR alpha and beta chain repertoires. After RT, the cDNA was added poly C at the 3`end and then used as the next PCR templates. The forward Abridged Anchor Primer (5’-GGCCACGCGTCGACTAGTACGGGIIGGGIIGGGIIG -3’) and the corresponding reverse biotinylated primers (Primers 11–20, **S1 Table**) were used to the amplification of TCR and BCR. Next, the PCR products were subjected to sonication by the Covaris S220 (Covaris, Massachusetts, USA.), and then 150-250bp DNA band was gel-purified out. These purified products were further screened by the streptavidin magnetic beads (Dynabeads M-270, Invitrogen, California, USA) for acquiring our target DNA (the region indicated by two dotted lines, Procedure 6–7, **S1 Fig**). Finally, the screened DNA was used for the Hiseq library construction and it could be referred to the manufacturer’s protocol (Illumina, San Diego, USA). We adopted the pair-end 101 strategy to sequence the CDR3 repertoire of the CR.

### Data analysis

In brief, we got 49 TRAV and 60 TRAJ genes from the GJ germline gene database, we then filtered those genes in which we cannot find the conserved C amino acid at the 3’ end of V gene (here we define the last 30 bases of the V terminal as 3’ end) or the conserved [FW]GXG motif in J gene according to the definition of CDR3 which starts from the last cysteine of V gene and ends at the phenylalanine in the J gene motif [F/W]GXG. After the filtering, we obtained 31 TRAV and 55 TRAJ genes and these V and J genes were used into the sequence alignment.

Paired-end sequencing strategy with 100 bp length was adopted to capture the entire CDR3 of TCR and BCR using illumina hiseq 2000 platform. The sequencing raw data was processed using our self-developed tool, IMonitor [25]. Specifically, the IMonitor pipeline contains an error correction step to reduce the impact of the PCR and sequencing errors on the downstream analysis as much as possible [26]. In brief, the data processing procedures of IMonitor mainly includes the following several steps: first, all of raw reads were treated with a quality control procedure in which, for example, reads with an average quality score lower than 15 or with more than 5% N bases were removed. Next, those reads that passed filtering were then merged into one sequence using FqMerger (a small program embedded in IMonitor that was used to do the first-step merging for pair-end reads, implemented by the Java language) and the COPE [27]. Second, these merged sequences were aligned to the corresponding reference genes using a local BLAST program (version 2.2.25) and each sequence was assigned an optimal V and J genes [28]. Those sequences that aligned with a non-functional V or J gene were filtered. Finally, we extracted CDR3, V/J and V-J pairing usage, and other useful information and performed downstream analysis based on sequence alignment information.

In order to separate the IGK and IGL sequences from mixed IGKL sequences, we aligned the mixed IGKL sequences with IGK and IGL reference genes respectively and extracted the IGK and IGL repertoire sequences based on the alignment information of each sequence. All raw sequence data has been uploaded into the Sequence Read Archive (SRA) repository of the NCBI under the accession number PRJNA389234.

### Statistical analysis

Rarefaction analysis was developed in 1968 by Howard Sanders in a biodiversity assay of marine benthic ecosystems [29]. Now, it is one of the most commonly used method in ecology to assess species richness from the results of sampling. The Chao 1 estimator is a non-parametric method that is used to calculate the true species diversity of a sample by the equation [30]:

$$S_{1}=S_{obs}+\frac{F1^{2}}{2*F2}$$

where S_obs_ is the number of distinct CDR3 amino acid sequences in a repertoire, F1 is the number of distinct CDR3 amino acid sequences that with only a single occurrence in the repertoire, and F2 is the number of distinct CDR3 amino acid sequences that with exactly two occurrences in the repertoire.

We calculated the value of S_obs_ and S_1_ at 10 different points and at each point we randomly repeated 10 times. In brief, at the first point, we randomly took 10 percent of total reads to calculate the value of S_obs_ and S_1_, and then 10 times random repetitions were performed at this point. At the next point, we randomly took 20 percent of total reads and calculated the value of S_obs_ and S_1_, and as the same as at the first point, 10 times random repetitions were performed, the following and so on. Using these calculated S_obs_ and S_1_, we drawn the rarefaction curve to see whether we had captured the majority diversity of a specific repertoire.

## Results

### Data description

We obtained about 3.30, 3.36, 2.15 and 2.48 million pairs of raw reads from TCRA, TCRB, IGKL and IGH repertoires, respectively. After the processing of IMonitor pipeline, about 1.16, 1.09, 0.48 and 0.30 million productive sequences were retained, respectively. We then separated the IGK and IGL data from mixed IGKL data based on the alignment information respectively and obtained about 0.32 and 0.16 million productive sequences for IGK and IGL repertoires, respectively. Finally, we identified 87938, 218139, 53496, 40316 and 163405 distinct CDR3 nucleotide sequences from TCRA, TCRB, IGK, IGL and IGH repertoires, respectively. A detailed data description can be seen from **S2 Table**.

### The usage of functional V, J genes and V-J pairing

**Fig 1:**
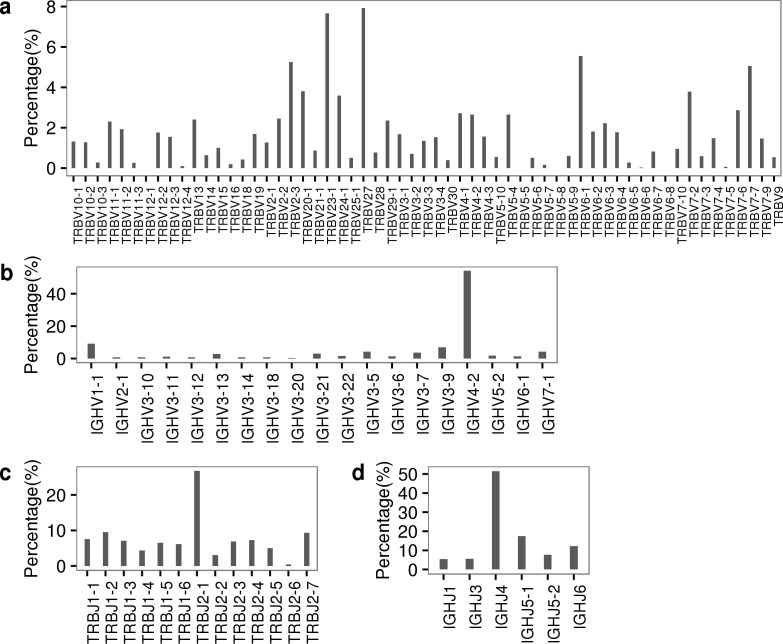
The usage frequency of each functional V/J gene for TCRB and IGH repertoires. (a-b) The usage frequency of each functional V gene in TCRB and IGH repertoires, respectively. (c-d) The usage frequency of each functional J gene in TCRB and IGH repertoire, respectively.

For each functional V and J genes in TCRA, TCRB, IGK, IGL and IGH repertoires, we calculated its relative usage frequency using the sequences that aligned best with it, respectively (**Fig 1, S3–S5 Tables**). We noticed that the usage of V/J gene showed a non-uniform distribution in rhesus macaque’s TCR and BCR repertoires. for example, the top three usages of V genes in TCRB repertoire accounted about 20% of total reads, whereas the last three usage of V genes only accounted about 3% of total reads. In addition, we also compared the usage of V genes between a previous published TCRB repertoire data that obtained from a single rhesus macaque based on Multiplex PCR method and our 5’ RACE -based data [20] (**S2 Fig)**. Both results were comparable but also showed some discrepancies.

We noticed that the frequency of IGHV4-2 was up to 55% in IGH repertoire, which was extremely high and made us suspect the accuracy of our result. We then looked through previous published studies to see whether this high usage of IGHV4-2 was caused by experimental bias or was indeed real in rhesus macaque’s IGH repertoire. It should be noted that the number of functional V genes of rhesus macaques in the IMGT database (n = 23) was disproportionately low compared to the human sequences (n = 53) [31]. Sundling et al. revealed 61 VH genes (termed the “CS germline gene database” hereafter) by mining rhesus macaque’s IGH locus from published rhesus macaque genome [32, 33]. Later, Dai et al. analyzed the usage of V genes in a rhesus macaque immunized with an HIV-1 trimer vaccine using both the CS germline database and the IMGT germline database, and they found that IGHV4-2 was dominant in IGH repertoire when IMGT germline database was used as reference, however, when the CS germline database was used, the most frequent V usage of V4-2 was around 10% [10]. Although they used a vaccine immunized rhesus macaque, however, previous studies had reported that HIV-1 vaccine induced broadly neutralizing antibodies (bNAbs) display a restricted VH gene usage of VH1-2*02 [34, 35]. Thus, the dominant usage of IGHV4-2 in our data was likely the real phenomenon in rhesus macaque’s IGH repertoire and was caused by the incomplete VH germline gene database.

**Fig 2:**
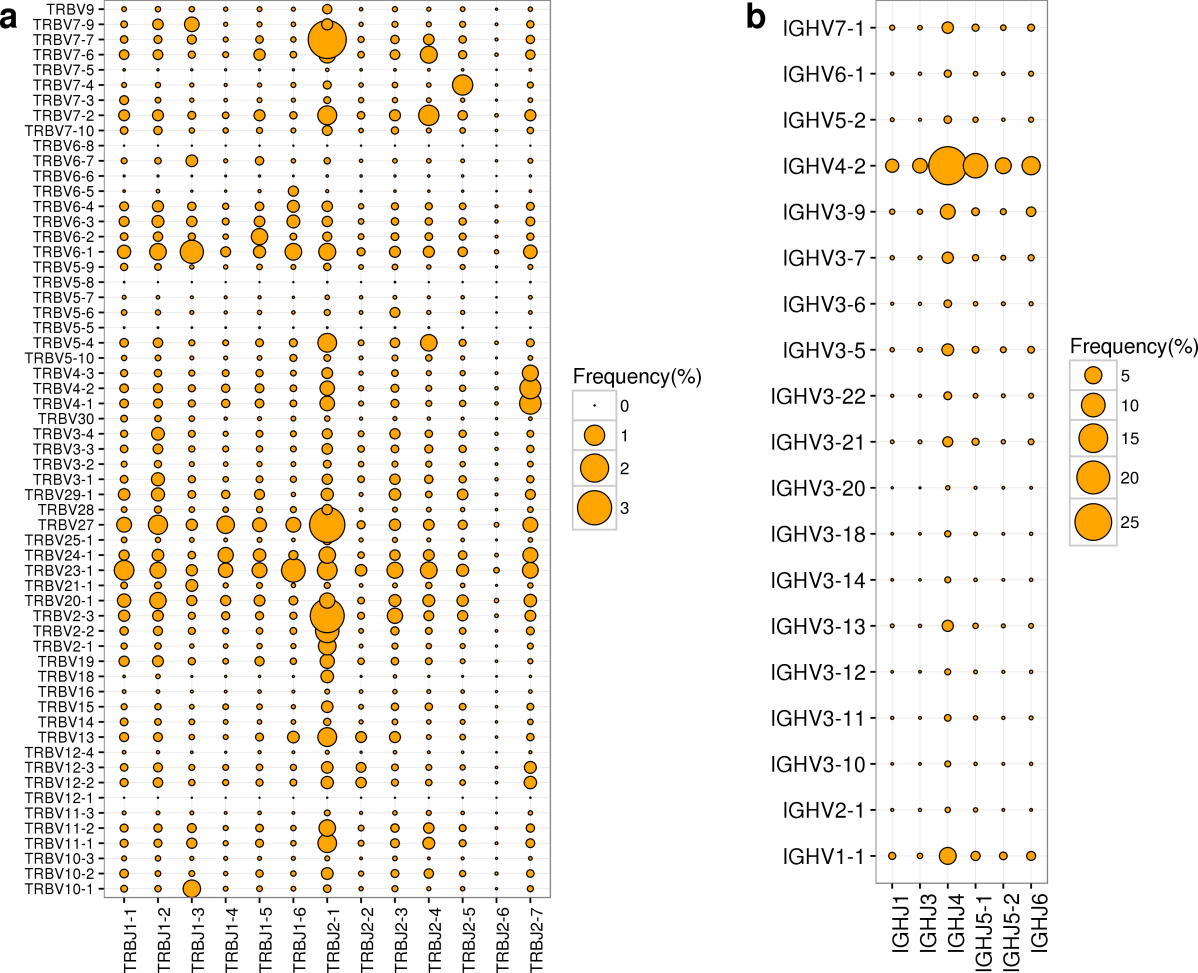
The usage frequencies of all possible distinct functional V-J pairing in TCRB and IGH repertoires. (a-b) The frequencies of all possible distinct functional V-J pairing in TCRB and IGH repertoires, respectively. The x axis represents all functional J genes and the y axis represents all functional V genes. The area of the circle is proportional to the frequency of a V-J pairing.

At present, the rhesus macaque has 59 TRBV and 13 TRBJ functional genes, 31 TRAV and 55 TRAJ functional genes, 83 IGKV and 4 IGKJ functional genes, 83 IGLV and 5 IGLJ functional genes, and 19 IGHV and 6 IGHJ functional genes. Therefore, there are 767, 1705, 332, 415, and 114 possible V-J combinations for TCRB, TCRA, IGK, IGL, and IGH repertoires, respectively. In our dataset, we successfully captured the majority of V-J combinations from TCRB (~95%), TCRA (~90%), IGK (100%), IGL(100%), and IGH (~99%) repertoires. Due to the unequal usage of V and J genes in TCR and BCR repertoires, we found that the usage of V-J combination in TCR and BCR repertoire were also non-uniform (**Fig 2, S6 Table**). In the TCRB repertoire, we found that the top three V-J combinations all had the TCRBJ2-1 gene (**Fig 2A**). Within IGH repertoire, the top three usages of V-J combinations were IGHV4-2/IGHJ4 (27.01%), IGHV4-2/IGHJ5-1 (10.70%) and IGHV4-2/IGHJ6 (5.74%) (**Fig 2B**).

### The length distribution of CDR3

**Fig 3:**
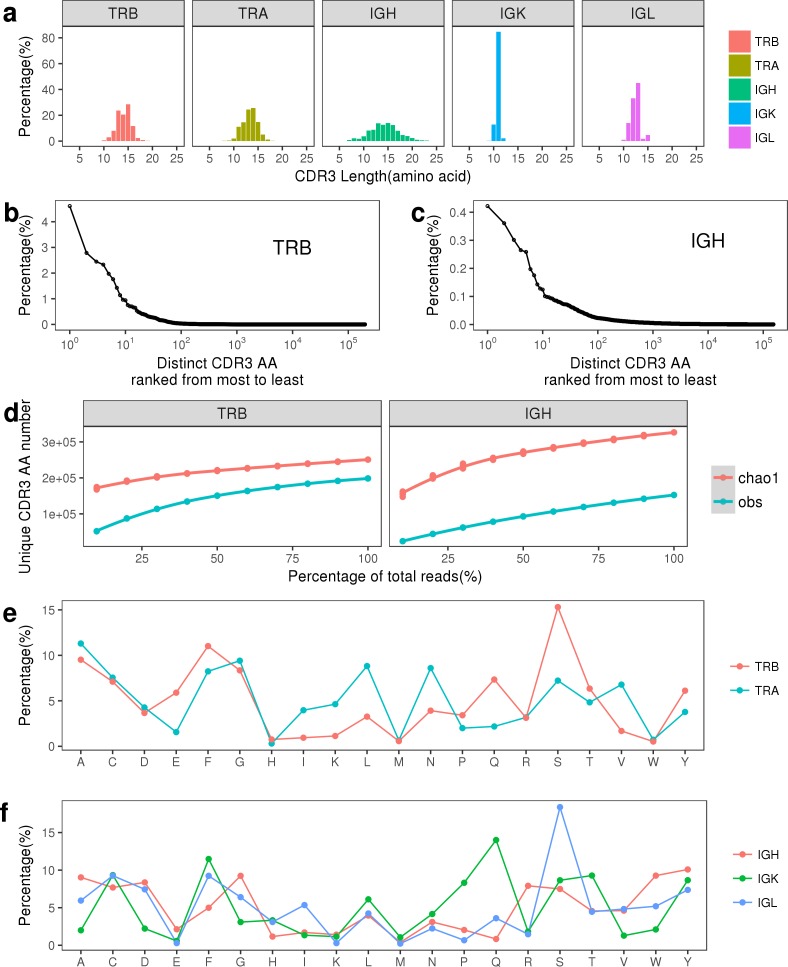
Several characteristics of CDR3 in TCRB and IGH repertoires. (a) The length distribution of CDR3 (at amino acid level, our CDR3 includes the first C and the last F/W). (b-c) The CDR3s were ranked from highest to lowest by their frequency in TCRB and IGH repertoires, respectively. (d) The rarefaction curve of CDR3 in TCRB repertoire (left) and IGH repertoire (right). (e) The composition of twenty kinds of amino acids of CDR3 in TCRA and TCRB repertoires. (f) The composition of twenty kinds of amino acids of CDR3 in IGK, IGL, and IGH repertoires.

The CDR3 region is formed by the V(D)J recombination and is highly variable in both TCR and BCR. In human, the length of the CDR3 (amino acid level) in both TCRB and IGH ranged mainly from 10 to 20 [4]. In rhesus macaque, we found that the length of CDR3 in both TCRB and IGH repertoires are comparable with that in humans (our CDR3 includes the first C and the last F or W) (**Fig 3A**). Within IGK repertoire, around 85% of CDR3 had 11 amino acids. Within IGL repertoire, around 33% of CDR3 had 12 amino acids and 45% of CDR3 had 13 amino acids. Thus, we can see that the CDR3 loop of IGL is slightly longer than that of IGK. We also found that the relative frequencies of majority distinct CDR3 nucleotides were less than 1% in TCRB repertoire (**Fig 3B**) and were less than 0.1% in IGH repertoire (**Fig 3C**).

### The potential number of distinct CDR3 and their amino acids composition

The CDR3 contacts directly with peptide-MHC complex, and a moderate diverse CDR3 pool can protect humans and animals from the invasion of foreign antigens. Previous studies have shown that within about 10 ml blood the number of CDR3 (amino acid level) in the rhesus macaque TCR repertoire (~260,000) is similar to the number of CDR3 in the human TCR repertoire (340,000) [20, 36]. To see if we had captured the majority of distinct CDR3, we used the rarefaction analysis to estimate the potential number of distinct CDR3 of TCR and BCR repertoires (see statistical method). The potential number of unique CDR3 predicted by Chao 1 estimator were 250790 in TCRB and 326427 in IGH, and the number of observed unique CDR3 were 198447 and 152444 in TCRB and IGH repertoires, respectively. Therefore, we successfully captured majority of CDR3 from TCRB repertoire (~79%), however, we failed to capture majority of potential CDR3 from IGH repertoire (only about 47% of potential CDR3 were captured), which implied that the sequencing depth in IGH repertoire was insufficient (**Fig 3D**).

We analyzed the amino acid composition of CDR3 and found that the frequencies of twenty kinds of amino acids varied a lot. For example, the amino acid frequency ranged from 0.30% (H) to 11.30% (A) within TCRA repertoire and ranged from 0.52% (W) to 15.30% (S) within TCRB repertoire (**Fig 3E**). Similarly, Hou X et al. found that the frequency of usage of amino acids within CDR3 intervals was remarkably consistent between ten SLE (Systemic Lupus Erythematosus) patients’ TCRB repertoires and the most frequently used amino acid was Serine (Ser, S), which accounted for 14.7% of all amino acids [37]. The feature of high usage of Serine between human’s TCRB repertoire and rhesus macaque’s TCRB repertoire needs further study. In addition, we found that there were six amino acids whose frequencies were comparable among BCRs’ three different chains (C, E, K, L, M and Y) (**Fig 3F**).

### Inserted and deleted nucleotides at junction

**Fig 4:**
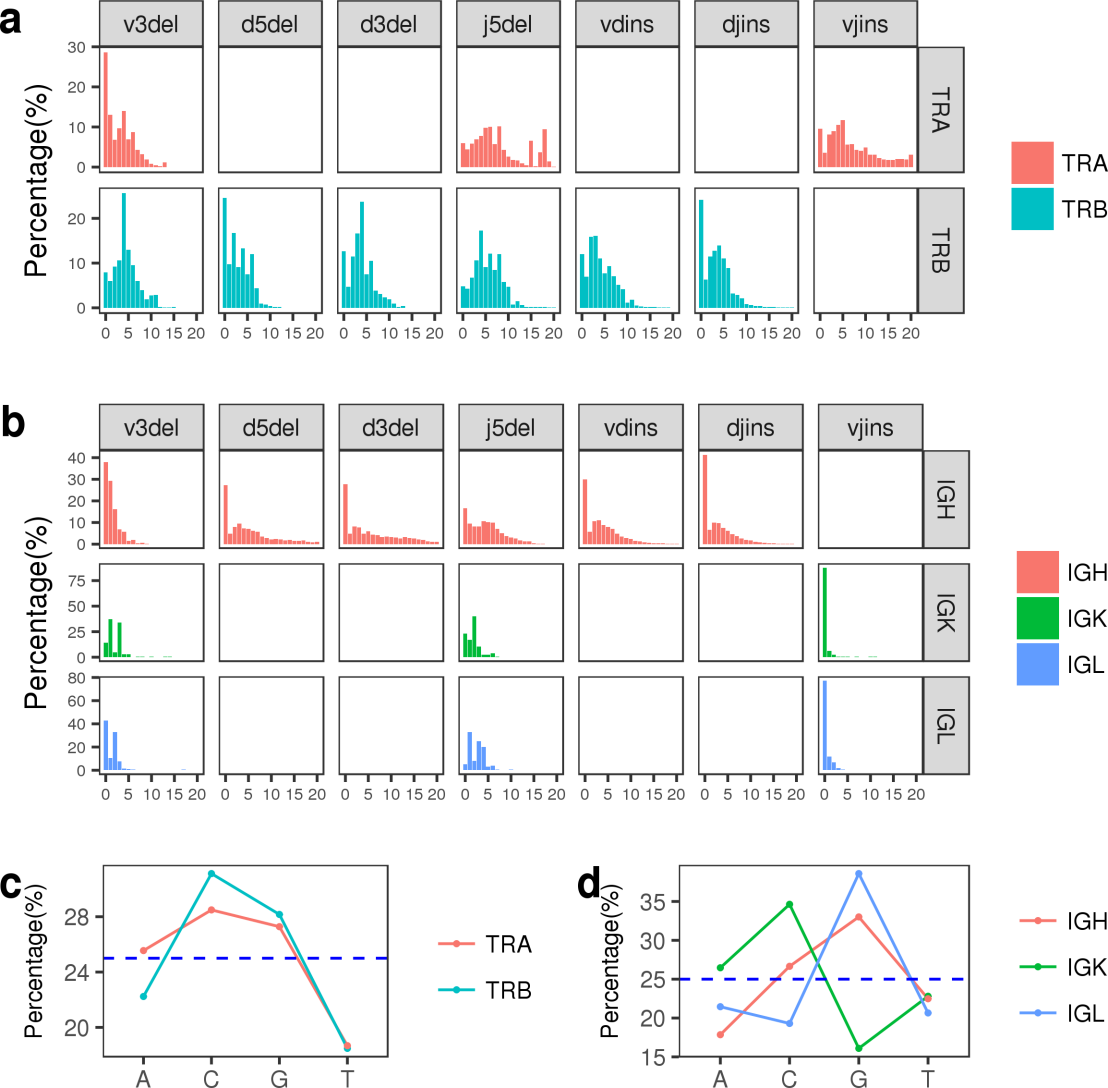
The insertion and deletion information at junctional regions. The junctional regions were divided into seven separate sections: 3’-V deletion, 5’-D deletion, 3’-D deletion, 5’-J deletion, V-D insertion, and D-J insertion sections. (a-b) The length distribution of inserted and deleted nucleotides at seven sections of junctional regions in TCRA/TCRB repertoires and IGK/IGL/IGH repertoires, respectively. The x axis represents the number of nucleotides. (c-d) The frequencies of four kinds of inserted nucleotides in TCRA/TCRB repertoires and IGK/IGL/IGH repertoires, respectively.

The recombination of V(D)J germline genes generates the primary diversity of TCR and BCR reperotires, however, this primary diversity is far from enough to generate various TCR and BCR. In fact, most of the diversity are derived from the template-independent insertions and deletions of nucleotides at V-(D)-J junctional sites by the terminal deoxynucleotidyl transferase [38]. Here, we calculated the length distribution of deletions at 3’ end of V gene, both ends of D gene, and 5’ end of J gene, as well as the length distribution of insertions at V-D, D-J and V-J junctional sites (**Fig 4A and 4B**).

A previous study reported that the inserted nucleotides at V-(D)-J junctional sites were biased to C and G in human TCRB repertoire [37]. Here, we calculated the frequencies of four kinds of inserted nucleotides at V-(D)-J junctional sites for all chains of TCR and BCR. Consistent with the previous results, the inserted nucleotides at V(D)J junctional sites in our data were also biased to C and G in TCRB repertoire (~31% C and ~28% G) (**Fig 4C**), and the same preference was also found in IGH repertoire (~27% C and ~39% G) (**Fig 4D**). However, the same insertion preference was not observed in IGK or IGL repertoire, on the contrary, the frequencies of inserted C and G varied greatly in both IGK and IGL repertoires (**Fig 4D**).

### Mutation analysis of the FR3

Unlike T cells, the naïve B cells will go through somatic hypermutation (SHM) when they encounter foreign antigens [39, 40]. SHM is a process of stepwise incorporation of single nucleotide substitutions into the variable region, and those B cells bearing the increased affinity of BCR will undergo preferential expansion [41]. Thus, the mutation analysis of BCR will help us understand the B cell differentiation and diversification. The SHM rates are not equal at different positions of variable region, and the frequencies of SHM in CDRs (CDR1 and CDR2) are relative higher than that in FRs (FR1-3) [42]. CDR3 is generated by the recombination of V(D)J germline genes and thus does not have its corresponding reference sequence, so we are unable to analysis the SHM of CDR3. besides, due to the 100 bp paired-end sequencing strategy, we found that most of our sequences can reach the FR3 region (from constant region to variable region), but failed to reach at the CDR2 (**S3 Fig**). Thus, the mutation analysis of BCR in this study was limited to the FR3.

According to the IMGT unique numbering system, the FR3 starts at the 196^th^ nucleotide and ends at the 310^th^ nucleotide in the V reference gene [43]. Based on this definition and the sequences alignment information, for each functional sequence, we can identify the mutated nucleotides, and at the same time, we can also determine the positions where the mutated nucleotides occurred. Having these information, we performed a detailed SHM analysis for the FR3 of BCR.

**Fig 5:**
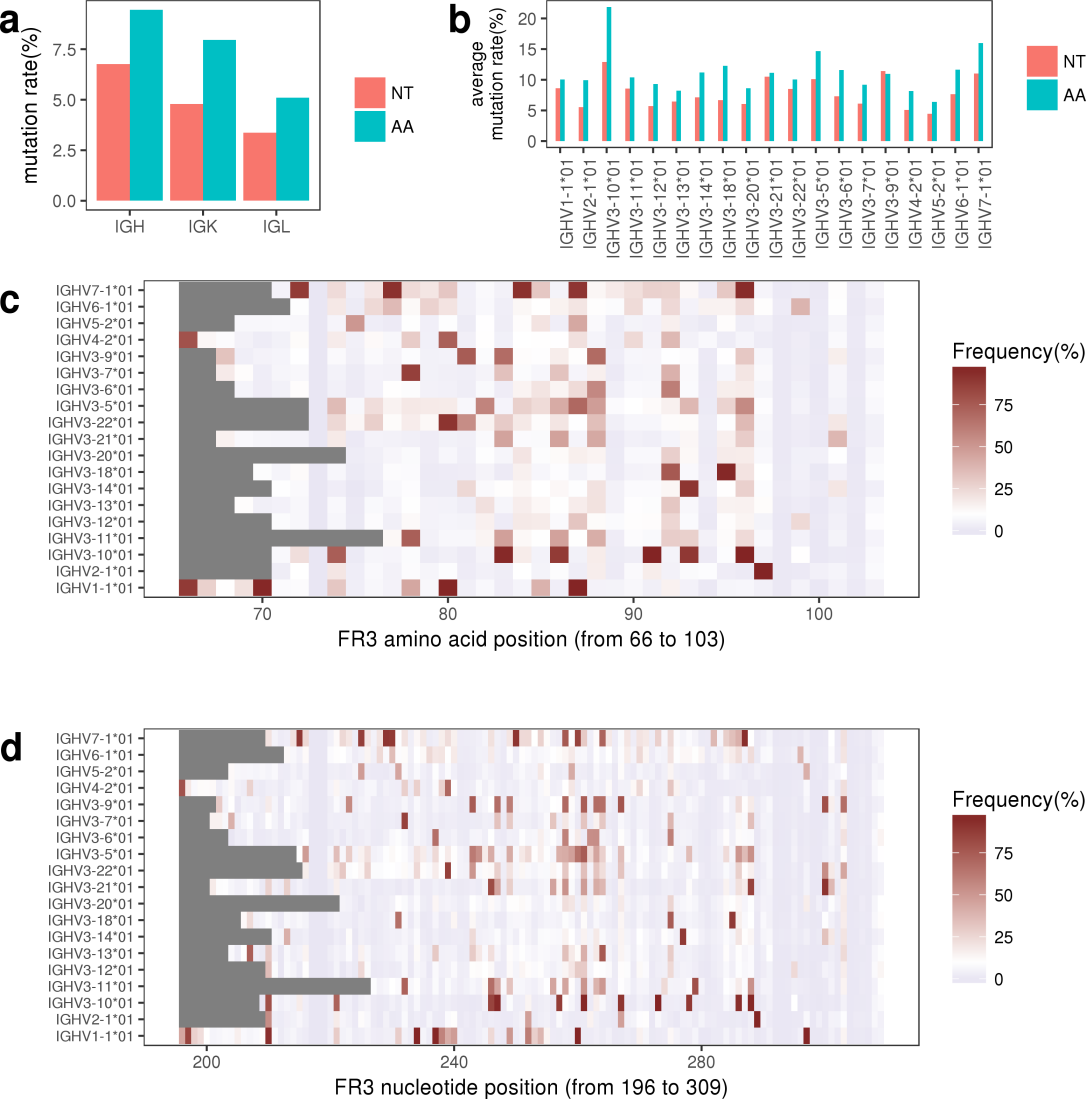
The mutation characteristics of FR3. (a) The overall mutation rates of FR3 in IGH, IGK, and IGL repertoires. NT represents the nucleotide and AA represents the amino acid. (b) The average mutation rates of FR3 among different functional V genes in IGH repertoire. (c-d) The distribution of mutation frequencies at each position of FR3 among functional V genes in IGH repertoire, at amino acid level (c) and nucleotide level (d). The grey rectangle indicates that no nucleotide was sequenced at that position or the number of nucleotide was less than or equal to 30 at that position.

First, we estimated the overall SHM rates of FR3 for IGK, IGL and IGH repertoires at both amino acid and nucleotide levels using all functional sequences. Here, the overall SHM rate is quantified as the number of mutated bases, divided by the number of all sequenced bases (considering the sequences abundance). On average, IGH repertoire had the highest SHM rate at both amino acid level (~8%) and nucleotide level (~6%) compared with other two types of light chain of BCR (**Fig 5A**).

Second, for each functional V gene, we calculated its average SHM rate of FR3 using those sequences that aligned with it (at amino acid and nucleotide levels). In IGH repertoire, we found the average mutation rates among different functional V genes ranged from ~6% to ~22% at amino acid level and ~4% to ~13% at nucleotide level (**Fig 5B**). In IGK repertoire, the average mutation rates among different functional V genes ranged between ~5% and ~27% at amino acid level and between ~3% and ~17% at nucleotide level (**S7 Table**). In IGL repertoire, the average mutation rates among different functional V genes ranged between ~2% and ~29% at amino acid level and between ~1% and ~17% at nucleotide level (**S7 Table**).

Next, we calculated the SHM rate for each position of FR3 base by base across each functional V gene. In brief, for each functional V gene, the sequences that best aligned with it were used to calculate the SHM of FR3 of this reference V gene. here, the SHM rate at a specific position of FR3 of a functional V gene is quantified as the number of mutated bases, divided by the number of sequenced or detected bases at that position [44, 45]. We noted that for a given reference V gene, the number of bases sequenced at different position varied widely. if the number of bases sequenced at one position is low, then we will not believe the SHM rate of this position at a very high confidence due to the sequencing and PCR errors. We used the IGHV3−22*01 gene to illustrate this problem. All sequences that aligned with IGHV3−22*01 gene were used for the SHM analysis (**S4 Fig**). S4A Fig shows the number of bases detected at each position of the FR3 of IGHV3-22*01 gene (blue line) and the number of bases that differs from the reference base at that position (red line). We can see that the number of bases detected at the 5’ end of the V gene was relatively small, compared with the number of bases detected on the 3’ end of the V gene. S4B Fig shows the SHM frequency at each position of the FR3 of IGHV3-22*01 gene, and we clearly found that although several positions had small number of detected bases at the 5’ end of V gene but had high mutation frequencies (**S4C Fig**). These positions are not likely to be credible and thus in the following analysis, we filtered out those positions where the number of bases is less than 30 to ensure the accuracy of SHM analysis. After the filtering, we displayed the FR3 SHM information (at nucleotide and amino acid levels) among different V genes with heatmap (**Fig 5C and 5D, S5 and S6 Figs**). We defined a position with mutation frequency greater than 10% as a mutation “hotspot” [46], and according to this definition we can see from Fig 4C (at amino acid level) and Fig 4D (at nucleotide level) that each functional V gene had a unique SHM “hotspot” feature that these hotspots occur at different positions of FR3 among different V genes. At nucleotide level, the number of SHM hotspot ranged from 8 (IGHV5-2) to 44 (IGHV7-1) in IGH repertoire (**Fig 5D**). In addition, we calculated the relative frequency for each mutation type within one repertoire and then compared the percentage of different mutation types among IGH, IGK and IGL repertoires at nucleotide level (**S7 Fig**). The top 15 most frequent mutation types in IGH, IGK and IGL repertoires were displayed in S8 Fig (at amino acid level).

A very interesting finding is that we found several positions not only with large number of detected bases, but also at the same time with high mutation frequencies in the IGHV3-22*01 gene. for example, the positions 300 and 303. When further analysis was performed, we were surprised to find that the mutated nucleotide (differs from the reference base at that position) at positions of 300 and 303 were mainly composed of only one type (300th position: 45.13% reference T and 54.42% mutated C; 303th position: 55.28% reference C and 36.06% mutated G) (**S4D Fig**). In general, the SHM frequency at the FR of V gene is lower than 10%, but in our data, the mutation frequencies at positions 300 and 300 are extremely high. Thus, we infer that the IGHV3-22 gene has polymorphic sites at positions 300 and 303, that is, in other words, the IGHV3-22 gene has other unreported alleles except the IGHV3-22*01 allele. This inferring is based on the following reasons: first, in consideration of the close genetic background between humans and rhesus macaques and the relatively small number of IGHV genes and/or alleles in rhesus macaques compared with humans, the existing IGH germline reference database of rhesus macaque is likely not complete; second, the genome of rhesus macaque is diploid, and a single rhesus macaque’s genome contains up to two different alleles for a certain V gene. If these two alleles were equally transcribed and sequenced at the same chance, then the number of base of two different nucleotides at a polymorphic site will be the same, which means that the frequency of mutated nucleotide at a polymorphic site is around 50%. However, due to the differences in the transcription level of the two alleles and the deviations in the library construction and sequencing process (for BCR repertoire, there exists SHM), the number of sequences corresponding to the two alleles may vary slightly. Thus, the frequency of mutated nucleotide at a polymorphic site may vary slightly. Considering the somatic mutation, if a mutated nucleotide at a specific polymorphic site fluctuates about 10% (40%~60%), then we can be very confident that this site is a potential polymorphic site for that V gene. Based on this criterion, we found 4 polymorphic sites for IGHV3-22 gene using IGHV3-22*01 allele (positions 239, 243, 300, and 303). The more polymorphic sites might be found for IGHV3-22 gene if more alleles of IGHV3-22 gene are used.

## Discussion

In this study, we did a deep profiling of a Chinese rhesus macaque’s immune repertoire using 5’ RACE method for the first time, including TCR and BCR. A comprehensive analysis including V, J and V-J pairing usage patterns, CDR3 length distribution, the composition of amino acids and nucleotides of CDR3, the distribution of inserted and deleted nucleotides at junction and mutation patterns were precisely performed.

We found that the usage of V, J and V-J pairing in rhesus macaque was unequal, and some V and J gene segments were preferentially used in T and B cells repertoires. for example, the TRBJ2-1 was widely used in many frequent V-J pairing of TCRB repertoire. we also found the unusual usage of IGHV4-2 gene in IGH repertoire, in which it accounted for about 50% of total reads, was resulted from the incomplete IGH germline gene reference in IMGT database. Until now, except the available CS germline database and IMGT germline database of rhesus macaque, the King’s College RM Ig Gene Database (http://www.kcl.ac.uk/immunobiology/Mac_ig/) also complied several other IGH germline genes that were reported from several other studies [16, 47, 48]. However, Guo et al. found that these RM VH genes from multiple sources existed substantial sequence identities and redundancies [31]. Thus, it is urgent to establish a non-redundant IGH germline gene database for rhesus macaque to improve the immune repertoire study on this model animal.

The SHM mainly occurs in CDRs (CDR1, CDR2 and CDR3) of variable region [42]. However, due to the limitation of sequencing length, we can only analyze the mutation information of FR3. We analyzed the mutation frequency each position of FR3 base by base for each functional V gene and found that each functional V gene had its own mutation feature Besides, we found several potential polymorphic sites for IGHV3-22 gene using IGHV3-22*01 allele and this direct method can be used for other functional V genes to search potential polymorphic sites for that specific V gene. In the future, these identified polymorphic sites should be validated strictly by experiments to test the accuracy of this method.

In the further study, the full-length BCR sequences capturing method should be introduced to facilitate the construction of precise lineage trees in mutation analysis. In summary, we report a comprehensive immune repertoires of a Chinese rhesus macaque for the first time, including all types of the chains of T cells and B cells, and find that the number of distinct CDR3 in Chinese rhesus macaque is comparable with the number of distinct CDR3 in humans. We believe this comprehensive profiling of TCR and BCR repertoires of rhesus macaque we provided will be of substantial interest and help to the broad scientific communities.

## Supporting information

S1 Fig Schematic diagram of the library preparation protocol. 1. cDNA was acquired from the total RNA through RT-PCR, with the CH1 primers. 2. mRNA degradation by RNAasemix. 3. Adding polyC tail to the 3`end of the cDNA. 4. PCR amplification with the AAP (Abridged Anchor Primer) and biotin labeled CH1 primers. 5. Supersonic DNA degradation and 150-250bp DNA gel-purification. 6. Target DNA (Biotin labeled) purification by Streptomycin magnetic beads. 7. Illumina sequencing adapter ligation and barcode (NNNNNN) addition. The target DNA region was indicated by two dotted lines in the bottom, about 150-200bp. The brace region (the left dotted line indicated) represents the 5’ends of different length target DNA fragments that supersonic breaked. The annealing positions of the CH1 primers and biotin labeled CH1 primers were also marked, and the specific binding sites of the CH1 regions could be referred to the primer ID of the S2 Table.
(PDF)

S2 Fig The usage of TCRB V genes between two different rhesus macaques. M1 represents our data, and M2 represents the data that was produced by Li Z group.
(TIF)

S3 Fig The length distribution of sequence segment before the second conserved Cysteine of V genes. According to the IMGT unique numbering system, the sequence segment that locates before the second conserved Cysteine of V genes was extracted from each of our merged sequences.
(TIF)

S4 Fig Demonstration of the distribution of FR3 mutation frequency in IGHV3-22*01 gene/allele (a) The number of sequenced and mutated nucleotides at each position of FR3 region. The red dot represents the number of mutated nucleotides at that position, and the blue dot represents the number of sequenced nucleotides at that position. (b) The relative mutation frequency at each position of FR3. (c) Several positions with small number of sequenced nucleotides but had relative high mutation frequencies. (d) the positions 300 and 303 had high mutation frequencies and at the same time the type of mutated nucleotides at these two positions were dominated by only one mutation type, which accounted for about 40%~60% of all bases at that position.
(TIF)

S5 Fig The distribution of mutation frequencies at each position of FR3 among functional V genes in IGK repertoire. The grey rectangle indicates that no nucleotide was sequenced at that position or the number of nucleotide was less than or equal to 30 at that position.
(TIF)

S6 Fig The distribution of mutation frequencies at each position of FR3 among functional V genes in IGL repertoire. The grey rectangle indicates that no nucleotide was sequenced at that position or the number of nucleotide was less than or equal to 30 at that position.
(TIF)

S7 Fig The composition of distinct mutation types within a repertoire (nucleotide level). (TIF)

S8 Fig The top 15 frequent mutated amino acid types in IGH (left), IGK (middle) and IGL (right) repertoires. (TIF)

S1 Table The 5’-RACE primers. (XLSX)

S2 Table Statistics of sequencing data. (XLSX)

S3 Table The usage frequency of V/J gene in TRA repertoire. (XLSX)

S4 Table The usage frequency of V/J gene in IGK repertoire. (XLSX)

S5 Table The usage frequency of V/J gene in IGL repertoire. (XLSX)

S6 Table The usage frequency of V-J pairing in TRA, IGK and IGL repertoires. (XLSX)

S7 Table The average mutation rate for each functional V gene in IGK and IGL repertoires. (XLSX)
