# Supplementary material for: A comprehensive profiling of T- and B-lymphocyte receptor repertoires from a Chinese-origin rhesus macaque by high-throughput sequencing
Source: PLoS One. 2017 Aug 16;12(8):e0182733. doi: 10.1371/journal.pone.0182733 (PMC5559085; doi:10.1371/journal.pone.0182733)
Supplement: S1 Fig — 1. cDNA was acquired from the total RNA through RT-PCR, with the CH1 primers. 2. mRNA degradation by RNAasemix. 3. Adding polyC tail to the 3`end of the cDNA. 4. PCR amplification with the AAP (Abridged Anchor Primer) and biotin labeled CH1 primers. 5. Supersonic DNA degradation and 150-250bp DNA gel-purification. 6. Target DNA (Biotin labeled) purification by Streptomycin magnetic beads. 7. Illumina sequencing adapter ligation and barcode (NNNNNN) addition. The target DNA region was indicated by two dotted lines in the bottom, about 150-200bp. The brace region (the left dotted line indicated) represents the 5’ends of different length target DNA fragments that supersonic breaked. The annealing positions of the CH1 primers and biotin labeled CH1 primers were also marked, and the specific binding sites of the CH1 regions could be referred to the primer ID of the S2 Table. (PDF) [file pone.0182733.s001.pdf]

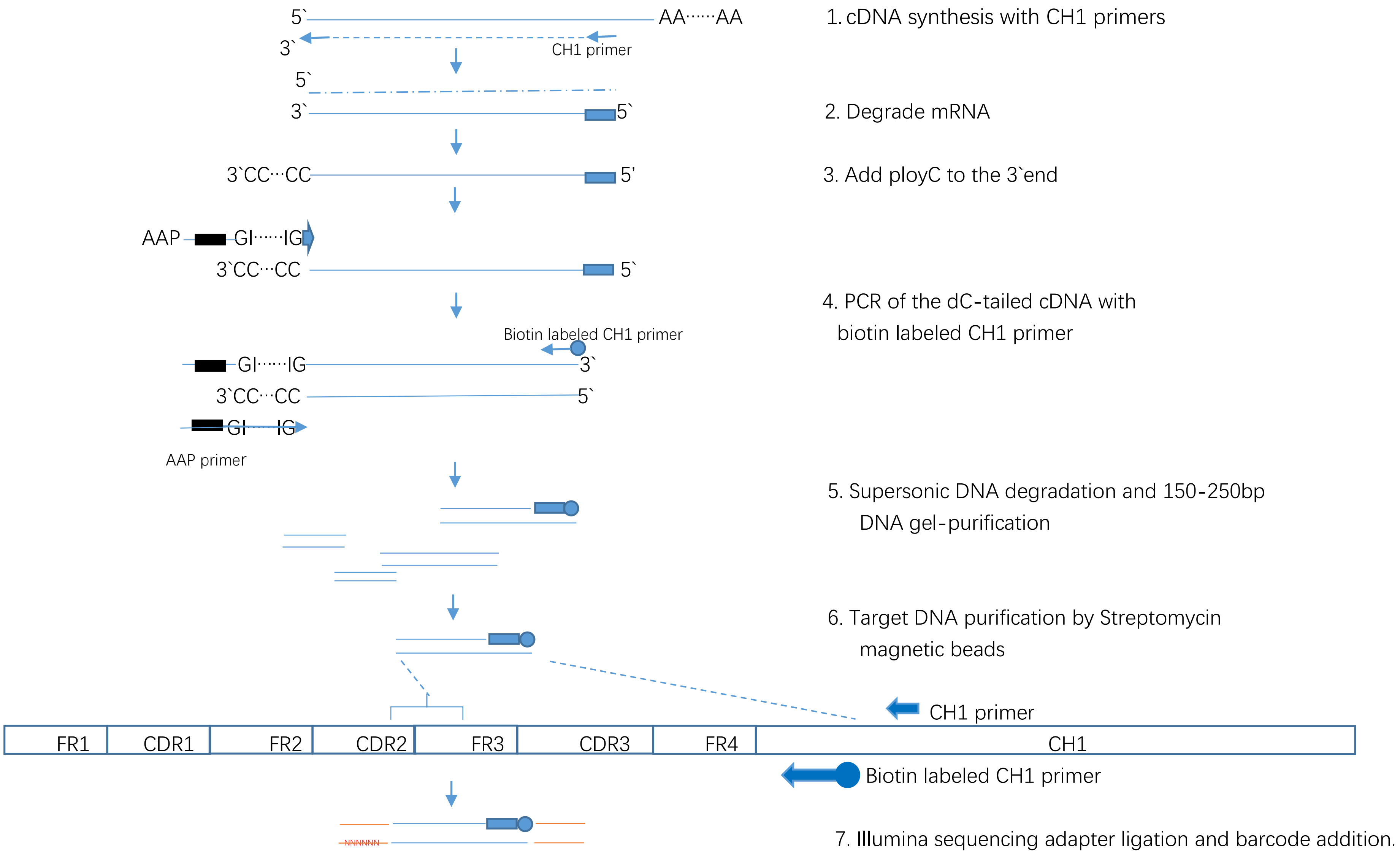
5`
AA······AA
3`
CH1 primer
5`
3`
5`
3`CC···CC
5'
AAP
GI······IG
3`CC···CC
5`
Biotin labeled CH1 primer
GI······IG
3`
3`CC···CC
5`
GI······IG
AAP primer
CH1 primer
FR1
CDR1
FR2
CDR2
FR3
CDR3
FR4
CH1
Biotin labeled CH1 primer
NNNNNN
1. cDNA synthesis with CH1 primers
2. Degrade mRNA
3. Add ployC to the 3`end
4. PCR of the dC-tailed cDNA with
biotin labeled CH1 primer
5. Supersonic DNA degradation and 150-250bp
DNA gel-purification
6. Target DNA purification by Streptomycin
magnetic beads
7. Illumina sequencing adapter ligation and barcode addition.
