# Supplementary figures and images for: A comprehensive profiling of T- and B-lymphocyte receptor repertoires from a Chinese-origin rhesus macaque by high-throughput sequencing
Source: PLoS One. 2017 Aug 16;12(8):e0182733. doi: 10.1371/journal.pone.0182733 (PMC5559085; doi:10.1371/journal.pone.0182733)

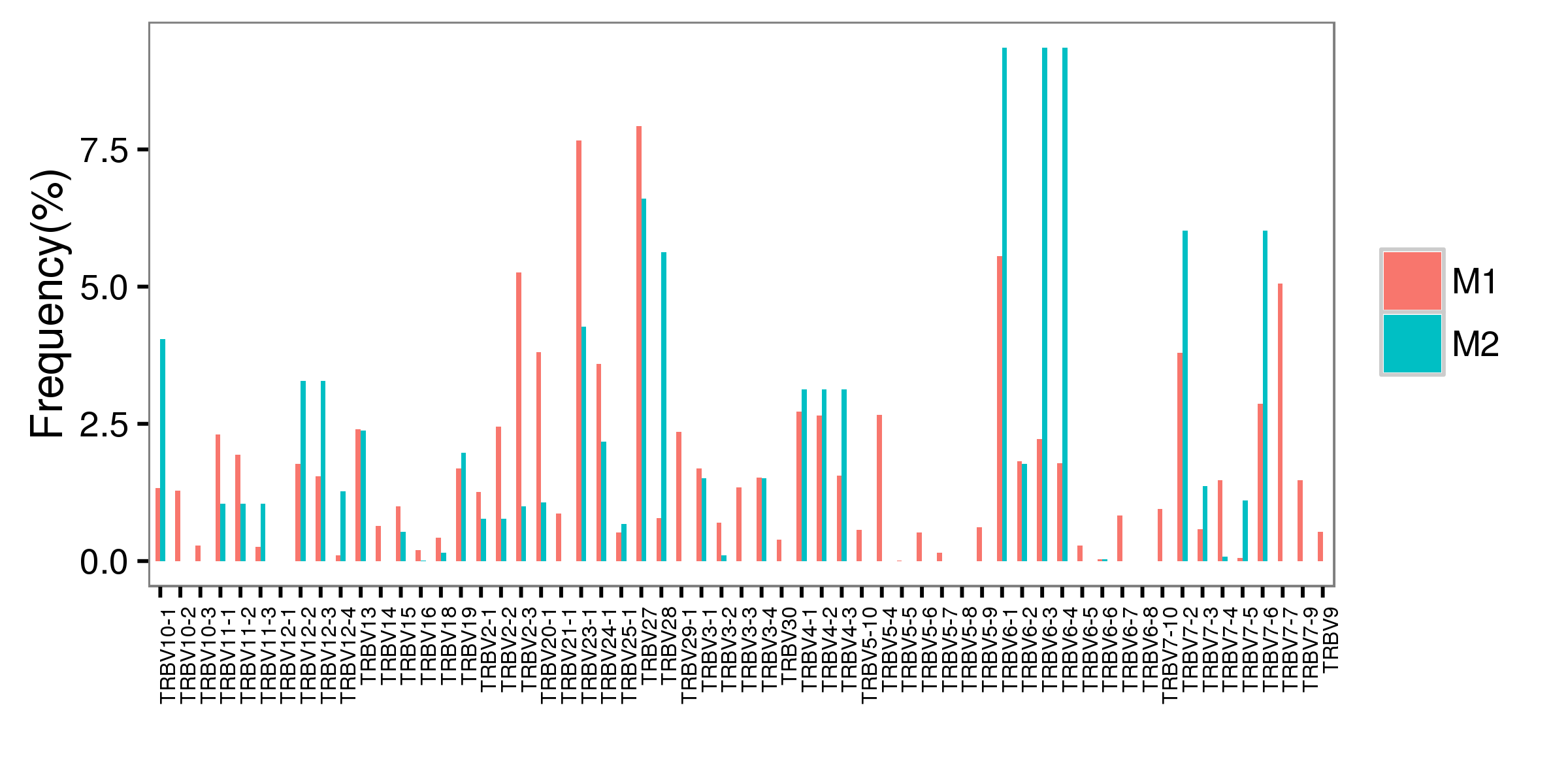

Supplement: S2 Fig — M1 represents our data, and M2 represents the data that was produced by Li Z group. (TIF) [file pone.0182733.s002.tif]

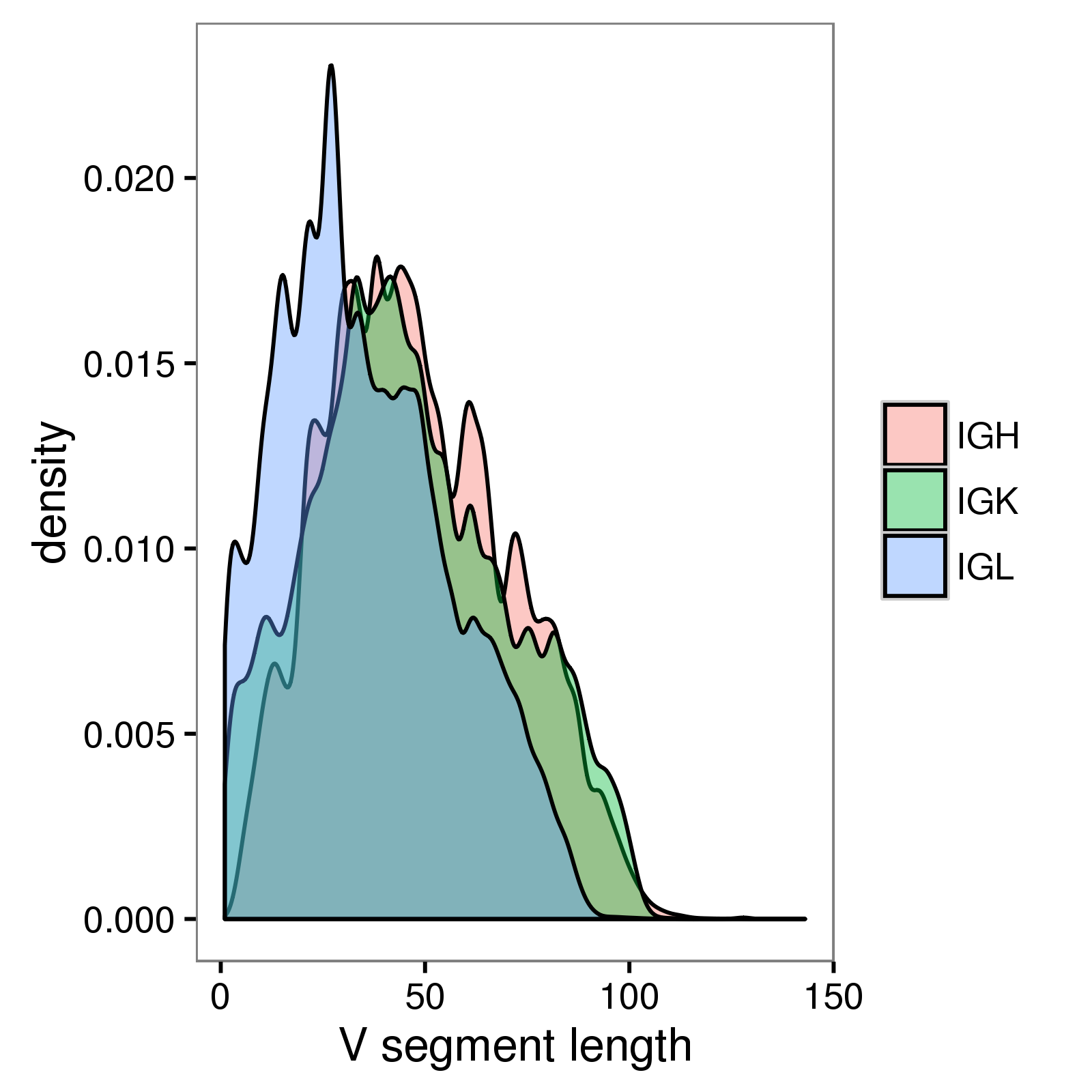

Supplement: S3 Fig — According to the IMGT unique numbering system, the sequence segment that locates before the second conserved Cysteine of V genes was extracted from each of our merged sequences. (TIF) [file pone.0182733.s003.tif]

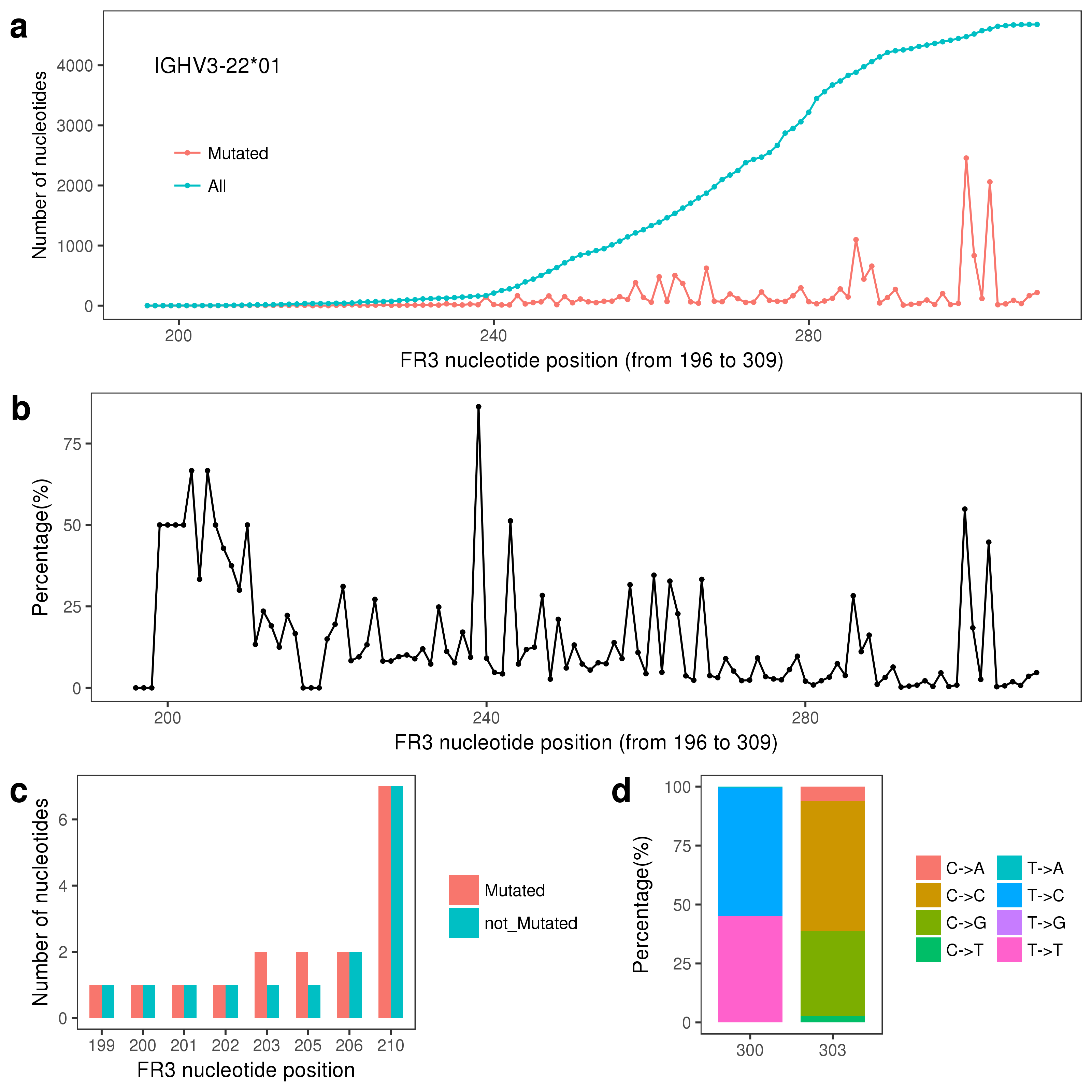

Supplement: S4 Fig — Demonstration of the distribution of FR3 mutation frequency in IGHV3-22*01 gene/allele (a) The number of sequenced and mutated nucleotides at each position of FR3 region. The red dot represents the number of mutated nucleotides at that position, and the blue dot represents the number of sequenced nucleotides at that position. (b) The relative mutation frequency at each position of FR3. (c) Several positions with small number of sequenced nucleotides but had relative high mutation frequencies. (d) the positions 300 and 303 had high mutation frequencies and at the same time the type of mutated nucleotides at these two positions were dominated by only one mutation type, which accounted for about 40%~60% of all bases at that position. (TIF) [file pone.0182733.s004.tif]

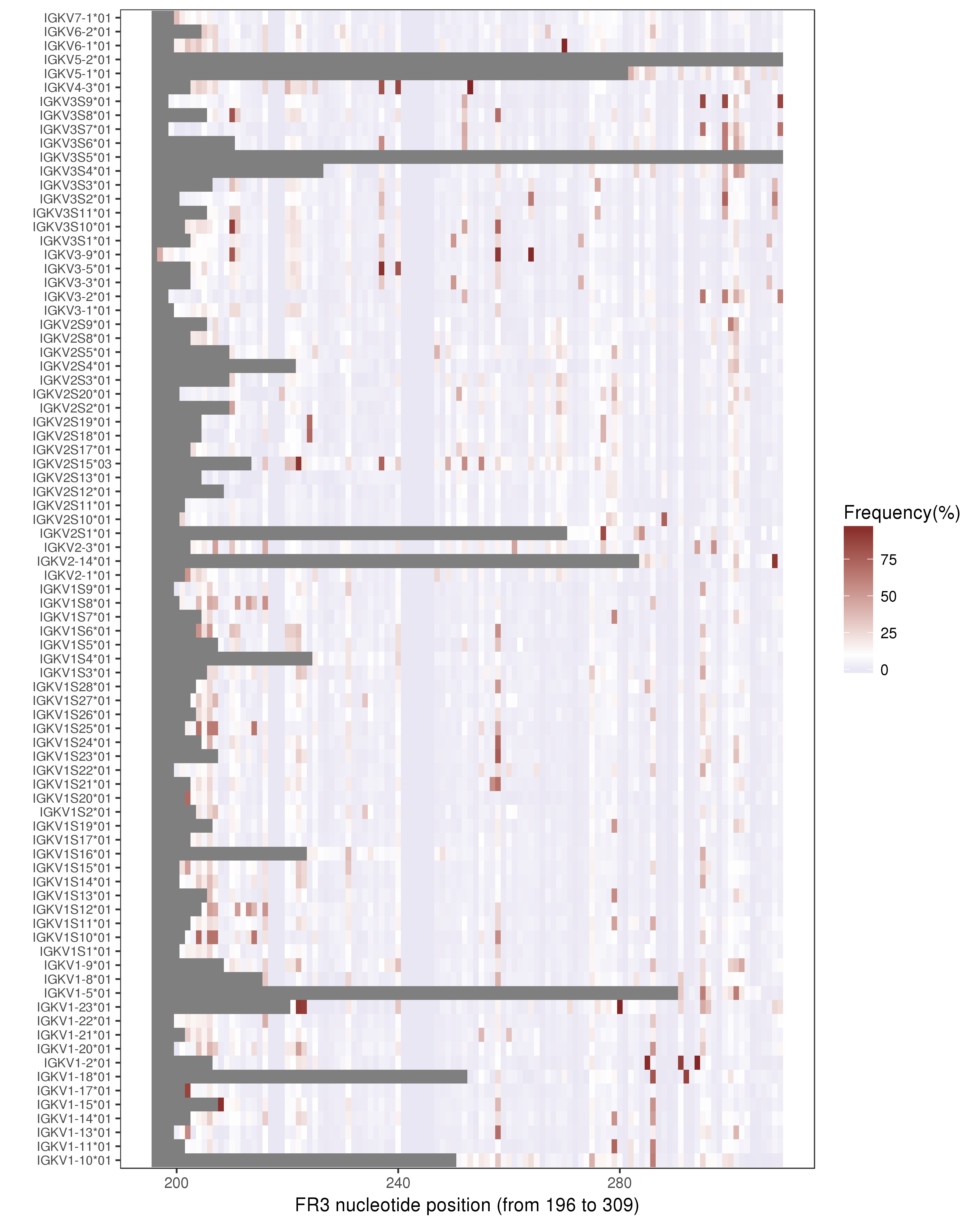

Supplement: S5 Fig — The grey rectangle indicates that no nucleotide was sequenced at that position or the number of nucleotide was less than or equal to 30 at that position. (TIF) [file pone.0182733.s005.tif]

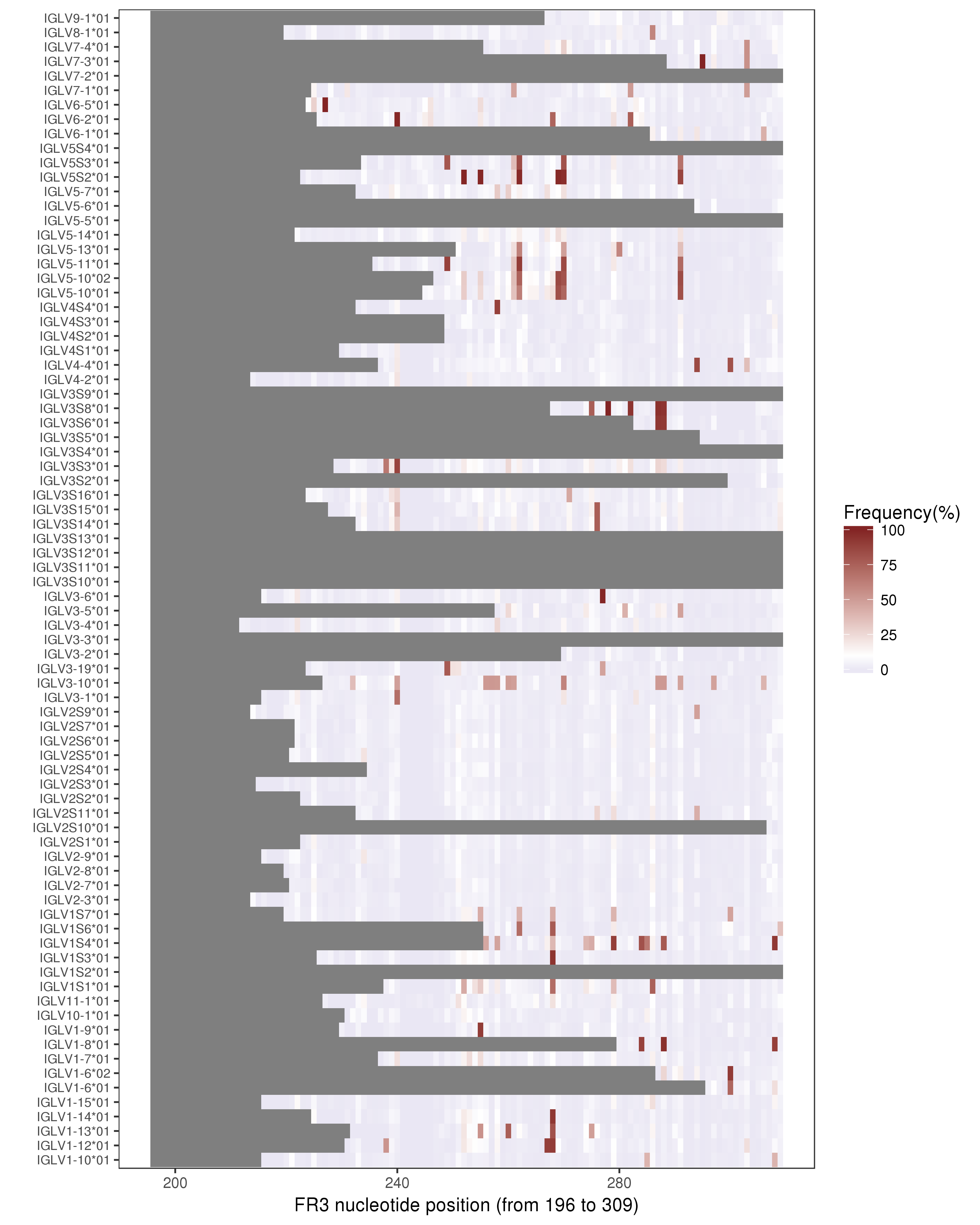

Supplement: S6 Fig — The grey rectangle indicates that no nucleotide was sequenced at that position or the number of nucleotide was less than or equal to 30 at that position. (TIF) [file pone.0182733.s006.tif]

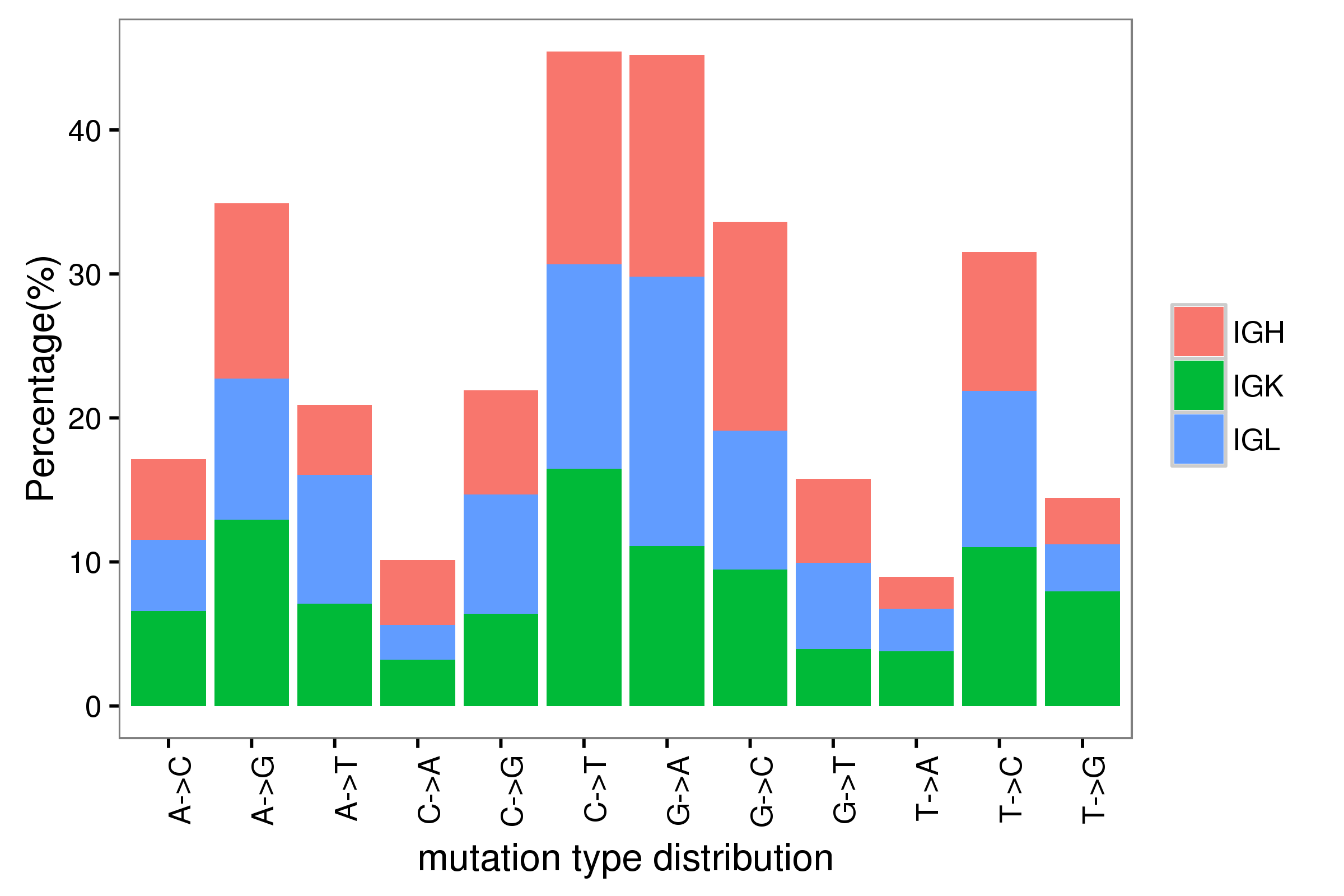

Supplement: S7 Fig — (TIF) [file pone.0182733.s007.tif]

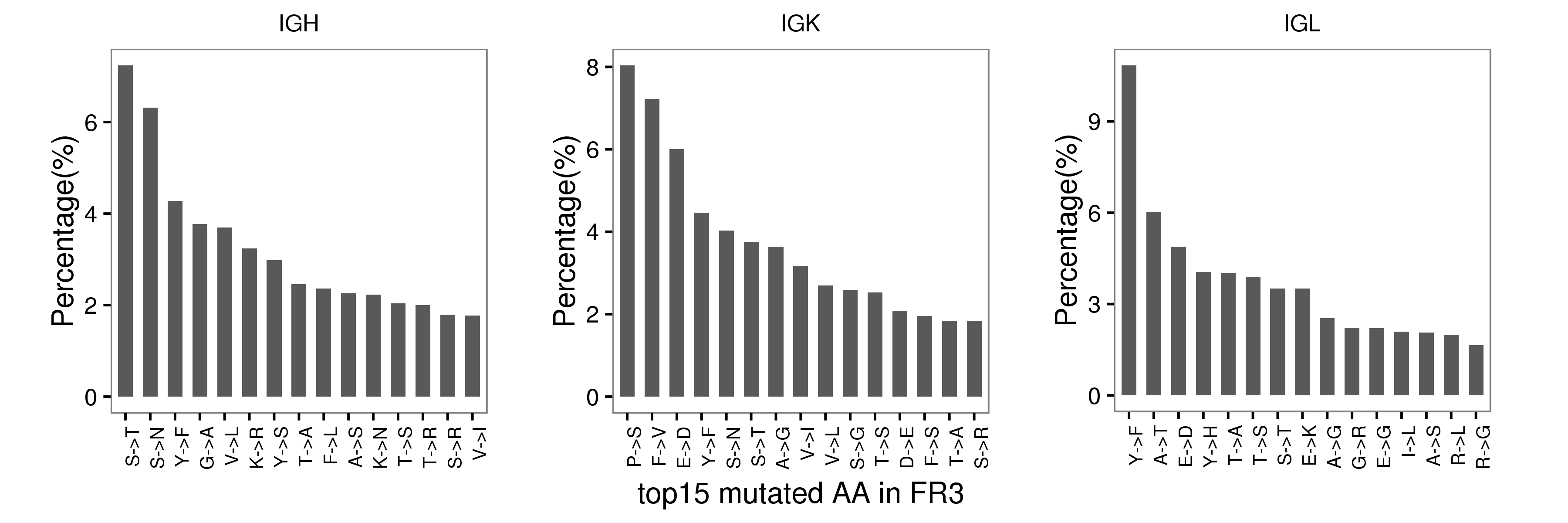

Supplement: S8 Fig — (TIF) [file pone.0182733.s008.tif]
